# Myocardial function including estimates of myocardial work in young adults born very preterm or with extremely low birthweight - a cohort study

**DOI:** 10.1186/s12872-023-03253-4

**Published:** 2023-04-29

**Authors:** Britt Engan, Tom R. Omdal, Gottfried Greve, Maria Vollsaeter, Elisabeth Leirgul

**Affiliations:** 1 Department of Clinical Science, University of Bergen, Bergen, Norway; 2 Department of Heart Disease, Haukeland University Hospital, Bergen, Norway; 3 Department of Paediatric and Adolescent Medicine, Haukeland University Hospital, Bergen, Norway

**Keywords:** Speckle-tracking echocardiography, Myocardial work, Very preterm, Extremely low birthweight

## Abstract

**Background:**

Preterm birth and low birthweight have been associated with increased risk of heart failure and cardiovascular disease in young adulthood. However, results from clinical studies of myocardial function are not consistent. Echocardiographic strain analyses allow detection of early stages of cardiac dysfunction, and non-invasive estimates of myocardial work can provide additional information on cardiac function. We aimed to evaluate left ventricular (LV) myocardial function including measures of myocardial work in young adults born very preterm (gestational age < 29 weeks) or with extremely low birthweight (< 1000 g) (PB/ELBW), compared with term-born age- and sex matched controls.

**Methods:**

63 PB/ELBW and 64 controls born in Norway in the periods 1982–1985, 1991–1992, and 1999–2000 were examined with echocardiography. LV ejection fraction (EF) and LV global longitudinal strain (GLS) were measured. Myocardial work was estimated from LV pressure-strain loops after determination of GLS and construction of a LV pressure curve. Diastolic function was evaluated by determination of the presence or absence of elevated LV filling pressure, including measures of left atrial longitudinal strain.

**Results:**

The PB/ELBW with mean birthweight 945 (standard deviation (SD) 217) grams, mean gestational age 27 (SD 2) weeks, and mean age 27 (SD 6) years had LV systolic function mainly within normal range. Only 6% had EF < 50% or impaired GLS >-16%, but 22% had borderline impaired GLS between − 16% and − 18%. Mean GLS in PB/ELBW (-19.4% (95% confidence interval (CI) -20.0, -18.9)) was impaired compared to controls (-20.6% (95% CI -21.1, -20.1)), p = 0.003. Lower birthweight was associated to more impaired GLS (Pearson correlation coefficient − 0.2). Means of EF, measures of diastolic function including left atrial reservoir strain, global constructive and wasted work, global work index and global work efficiency was similar in PB/ELBW and controls.

**Conclusion:**

The young adults born very preterm or with extremely low birthweight had impaired LV-GLS compared to controls, although systolic function mainly within normal range. Lower birthweight was associated with more impaired LV-GLS. These findings could indicate an elevated lifetime risk of developing heart failure in preterm born individuals. Measures of diastolic function and myocardial work were similar compared to controls.

**Supplementary Information:**

The online version contains supplementary material available at 10.1186/s12872-023-03253-4.

## Background

Due to improvement in neonatal intensive care over the last decades neonates born extremely preterm (< 28 weeks of gestation) now have survival rates > 80% [1–3], thus representing approximately 0.5% of all children growing up in Norway [4]. Preterm birth and low birthweight are associated with increased prevalence of cardiovascular risk factors [5] and a higher risk of cardiovascular disease, all-cause mortality, and young adult death [2, 6–11]. Gestational age (GA) and birthweight (BW) are found to be inversely associated with increased risk of cardiovascular disease and all-cause mortality [2, 11], but an increased risk has been described even in individuals born only moderate to late preterm (32–37 weeks of gestation) [2, 11].

Large register-based studies have found increased risk of heart failure in adolescents and young adults born preterm [8, 12], but clinical studies of myocardial function measured by echocardiography or cardiac magnetic resonance imaging have reported diverging results. Several studies have reported mainly preserved left ventricle (LV) ejection fraction (EF), but impaired LV myocardial strain, diastolic function and myocardial response to physical stress [13–17]. However, LV myocardial strain patterns have also been described as normal or even hypercontractile [18].

Traditionally, definitions of, and guidelines regarding management of heart failure have been based on measures of EF. In later years, echocardiographic speckle-tracking derived strain analysis has allowed detection of subclinical early stages of LV dysfunction, and its role in risk stratification is increasing [19]. Additionally, although not yet much used in clinical practice, analyses of non-invasively measured LV myocardial work (MW) to describe myocardial function have been introduced as an advancement of LV strain analyses, due to reduction of the strain analysis` load dependent limitations [20, 21]. These measures strongly correlate to invasively measured MW [22, 23]. New parameters describing diastolic myocardial function have also been introduced. Analysis of left atrial (LA) reservoir function measured by LA peak longitudinal strain has been reported to detect increased LV filling pressure and LV diastolic dysfunction at an earlier point than traditional echocardiographic diastolic parameters [24, 25] like mitral inflow and annular tissue velocities and LA volumes. Measures of LA reservoir function is reported to be of clinical and prognostic relevance especially concerning heart failure with preserved EF [26]. To our knowledge MW and LA strain have not yet been investigated in preterm born children or young adults.

We had the opportunity to investigate myocardial function in a cohort of young adults born very preterm or with extremely low birthweight, who have been followed since birth by the Project Extreme Prematurity in Bergen, Norway. The aim of this study was to investigate the impact of preterm birth and low birthweight on left ventricular myocardial function at young adult age, by adding new echocardiographic parameters such as MW and LA longitudinal strain to measures of LV EF, LV longitudinal strain and traditional echocardiographic measured markers of diastolic function.

## Materials and methods

### Study design and participants

The Project Extreme Prematurity, organized by the WestPaed Research group in Bergen, Norway, has included three regional cohorts of in total 153 young adults born very preterm or with extremely low BW (PB/ELBW), and in total 139 individually age and sex matched term-born controls. Inclusion criteria were birth at GA < 29 weeks or extremely low BW < 1000 g. Subjects born in 1982–1985 (cohort 1) and in 1991–1992 (cohort 2) were retrospectively recruited, while those born in 1999–2000 (cohort 3) were prospectively recruited. The inclusion and longitudinal follow-up of these cohorts have been described in detail previously [27, 28]. In total 6 individuals were lost to follow-up; 1 control and 2 PB/ELBW because of death and 3 PB/ELBW due to severe disability.

During the period 2017–2020 the participants were invited to a third follow-up, including a first-time echocardiographic examination. A total of 127 participants were examined, including 63 PB/ELBW subjects (43% men) and 64 controls (44% men) (See flow chart of study participation in Additional file 1). Of the participants, 49 belonged to cohort 1, 37 to cohort 2, and 41 to cohort 3.

The Regional Committee for Medical and Health Research Ethics of the Western Norway Health Authority approved the study (REC 2017/0068). Written informed consent was obtained from all participants.

### Echocardiographic assessment

All echocardiographic examinations were performed on a Vivid E9 ultrasound system (GE Healthcare, Horten, Norway) using a 5Sc (1.5–4.6 MHz) or equivalent 4Vc-D (1.4–5.2 MHz) transducer for all imaging. All images were saved as three consecutive cardiac cycles during sinus rhythm, on external hard drives allowing offline analyses using the echocardiographic software, EchoPAC version 204 (GE Healthcare, Horten, Norway). All participants underwent a comprehensive functional echocardiographic examination, which included grayscale images optimized for 2-dimensional (2D) speckle analysis. The images were analysed according to recommendations from the European and American Society of Echocardiography [29–31] and as proposed by Russel et al. [23] regarding analyses of MW.

LV EF was calculated using the modified Simpson biplane technique and from long-axis 2-dimentional (2D) measurements using the Teicholz formula. LV wall thickness, LV internal diameter and myocardial mass index (calculated using the Devereux formula and indexed to body surface area (BSA)) were calculated using long-axis end-diastolic 2D measurements. Diastolic function was evaluated as recommended by determination of the presence or absence of elevated LV filling pressure [31], and was measured by pulse waved Doppler (early/late mitral inflow velocity (E/A)), continuous wave Doppler (tricuspid valve regurgitation jet peak gradient), tissue Doppler (mitral septal and lateral annular early diastolic velocity (e´) and E/e´), and measures of LA end-systolic volume in four chamber view indexed by BSA.

Gray scale images for 2D speckle-tracking analysis were acquired at frame rate (frames/second) to heart rate (beats/minute) ratio 0.7–0.9. Longitudinal strain curves from one ventricular cycle from standard apical four, three and two chamber view images, analysed for 18 subsegments, were used for LV longitudinal strain assessment. For LA longitudinal strain assessment, we used longitudinal strain curves from one atrial cycle from apical four chamber view images, with zero reference point set at the start of the ECG R-wave.

Speckle-tracking of the LV and the LA were performed using automated function imaging (AFI). Visual inspection ensured that the region of interest (ROI) included the whole of the myocardium from the atrioventricular valve annulus to the LV apex or LA roof and from the endocardial border to the epicardial border. If the automatic tracking was not satisfactory, the ROI was adjusted manually until the best possible tracking was obtained. LV tracking compromised by artefacts/shadows or image acquisitions with less than five of six appropriately tracked segments per apical view were excluded. Peak systolic longitudinal strain was measured at aortic valve closure for LV measures and at end-systole for LA measures. MW was calculated by the EchoPAC software after determination of LV peak systolic global longitudinal strain (LV-GLS) and construction of a LV pressure curve. The LV pressure curve was derived from non-invasive brachial blood pressure (BP) measures and the timing of the cardiac cycles` isovolumetric and ejection phases, which were defined by opening and closure of the mitral valve. The area of the LV pressure–strain loop represents MW. Since work by definition equals force times length, the use of pressure and strain does not provide a direct measure of work, but can be used as a valid approximation of it as the method is found to strongly correlate to invasively measured MW [22, 23]. Global MW obtained from LV-GLS with less than 17 LV segments, due to suboptimal tracking or image quality, were excluded. The timing of the aortic and mitral valve opening and closure was decided using continuous-waved and pulse-waved Doppler, respectively, and confirmed by visualizing the opening and closure of the valves from the LV three chamber apical view.

**Fig. 1:**
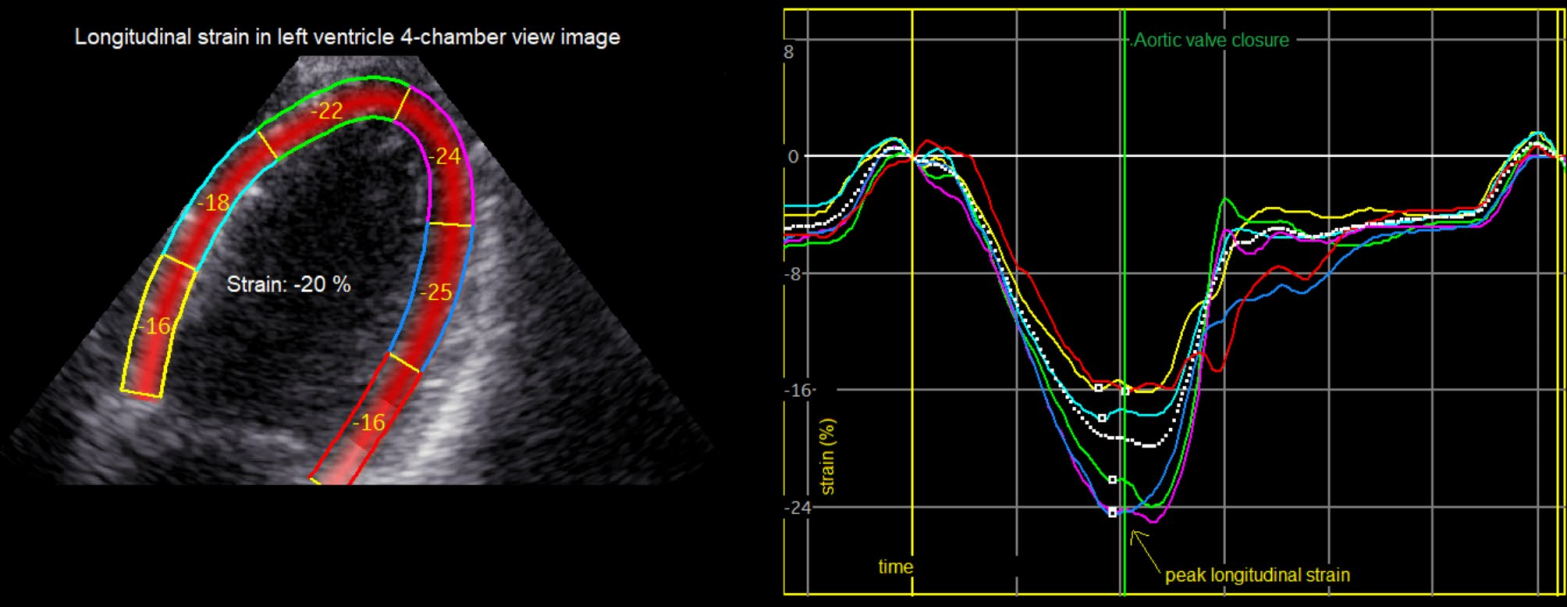
Left ventricle peak longitudinal strain measures generated from apical 4-chamber view image

In this paper, LV-GLS describes the average peak systolic longitudinal strain from all three left ventricular apical views, and LV-4 C-LS is used to describe the peak systolic LV longitudinal strain from the apical four chamber view (Fig. 1).

**Fig. 2:**
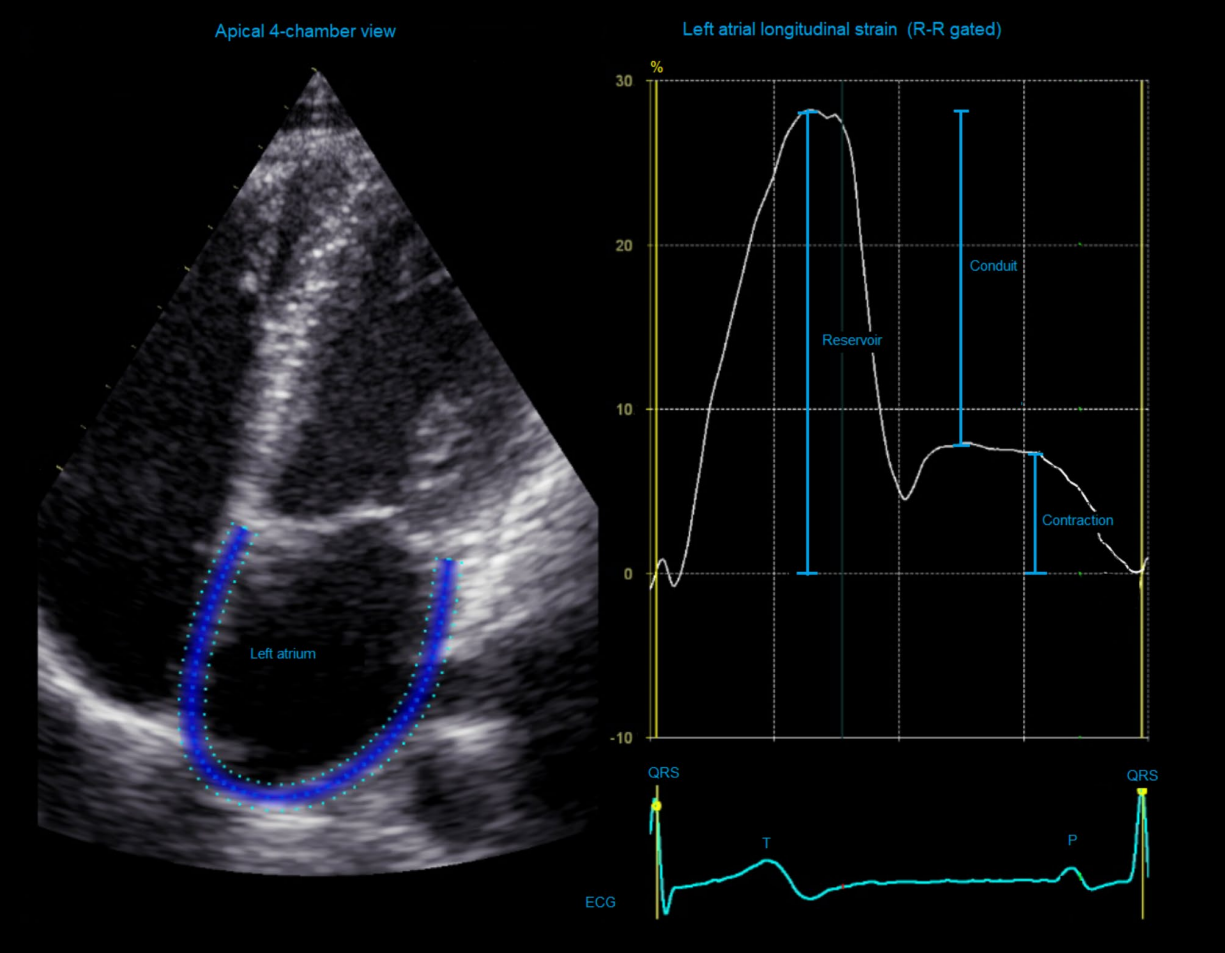
Left atrial reservoir, conduit, and contractile function measured by left atrial longitudinal strain in an apical 4-chamber view image

LA reservoir, conduit, and contractile strain generated from LA longitudinal strain analyses from the apical four chamber view images are used to describe LA reservoir function during LV systole, conduit function during early diastole, and booster pump function during late diastole (Fig. 2). All longitudinal strain values are described as negative percentages, as they describe myocardial shortening of segments relative to original length, except LA-reservoir and conduit strain which represent myocardial lengthening relative to original length and therefore are described as positive percentages.

**Fig. 3:**
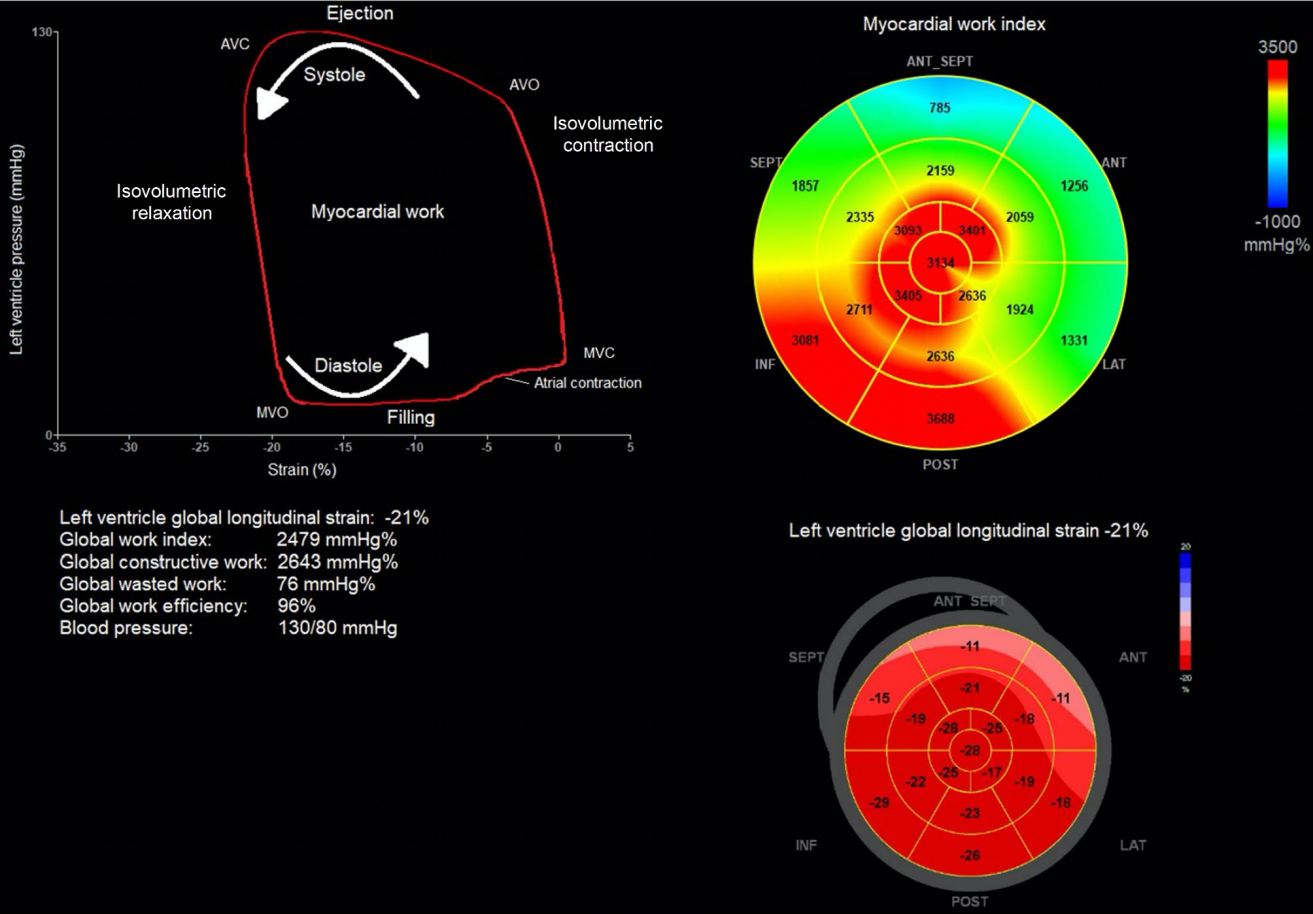
Myocardial work (MW) was calculated using EchoPAC version 204 (GE Healthcare) after determination of left ventricle (LV) peak global longitudinal strain and construction of a LV pressure curve. The LV pressure curve was derived from non-invasively measured brachial pressure and timing of the cardiac cycles` isovolumetric and ejection phase defined by mitral valve opening and closure. The area of the LV pressure–strain loop was defined as MW AVC: aortic valve closure, AVO aortic valve opening, MVO: mitral valve opening, MVC: mitral valve closure

Myocardial global constructive work (GCW), which contributes to ejection, is used to describe the mean net effect resulting from positive LV work (shortening) during systole plus negative LV work (lengthening) during isovolumetric relaxation in all 18 LV segments. Myocardial global wasted work (GWW), which does not contribute to ejection, is used to describe the mean net effect resulting from negative LV work during systole plus positive LV work during isovolumetric relaxation in all 18 LV segments. Global work index (GWI) is used to describe total performed LV work from the opening to the closure of the mitral valve, and global work efficiency (GWE) is used to describe global constructive work relative to the total of global constructive and waste work (GWE = GCW/(GCW + GWW)). MW values are described as pressure percentages (mmHg%) as they are derived from LV pressure-strain loop analysis (Fig. 3).

EF Simpson and EF Teicholz ≥ 50% were considered normal and EF < 50% considered reduced. LV-GLS >-16% was considered abnormal, -16% to -18% borderline normal, and <-18% normal [32, 33]. Abnormal values of conventional LV diastolic parameters were defined as septal or lateral e´ <0.07 m/s or < 0.10 m/s, respectively, average E/e´ ratio > 14 (using the average of septal and lateral e´), peak tricuspid valve regurgitation jet velocity peak gradient > 30 mmHg, and LA volume index > 34 ml/m^2^ /s [34].

Mean reference values of measures of MW are former described in the NORRE study as GWI 1896 mmHg%, GCW 2232 mmHg%, GWW 78.5 mmHg% and GWE 96% [21, 35].

Mean reference values of LA strain are previously described as 39% for reservoir strain, 23% for conduit strain, and − 17% for contractile stain based on findings in a meta- analysis which included both ECG p-wave (P-P gated) or QRS complex (R-R gating) as initiation of LA stain calculations [36].

All imaging and analyses were performed by a single experienced cardiologist (B.E). A random sample of 10 study participants were obtained for inter- and intra-rater analyses. Another experienced cardiologist (T.O) performed the re-analysis for inter-rater reliability. Both were blinded to group affiliation (PB/ELBW or control) and to the original results during re-analyses.

Inter-rater reliability measured by a 2-way mixed-effect model for absolute agreement was 0.8 (95% confidence interval (CI) 0.04, 0.9) for EF Teich, 0.9 (95% CI 0.5, 1.0) for LV-GLS, 0.8 (95% CI 0.0, 1.0) for GWI, and 0.8 (95% CI 0.1, 0.9) for LA reservoir strain. Intra-rater reliability measured by a 2-way mixed-effect model for absolute agreement was 0.8 (95% CI 0.2, 0.9) for EF Teicholz, 0.8 (95% CI 0.3, 1.0) for LV-GLS, 1.0 (95% CI 0.8, 1.0) for GWI, and 0.9 (95% CI 0.6, 1.0) for LA reservoir strain.

### Blood pressure

BP was calculated as the average of three measurements with an automated oscillometric device (Biolight BLT V6, Biolight Meditech Company, Guangdong, China) at rest in supine position.

### Statistical analysis

Descriptive variables are presented as numbers (%) and outcome data as means with 95% CI or standard deviation (SD). The Kolmogorov- Smirnov test was used to test the normality of the outcome variables, and independent samples t-test (with equal variance not assumed) or two samples Kolmogorov-Smirnov test was applied for comparison between groups, as appropriate. For comparison of descriptive data Chi-square test or Fisher`s exact test was applied, as appropriate.

Linear regression analyses were used to identify confounders. Analyses of covariance (ANCOVA) was used for comparison between PB/ELBW and controls for height, weight, and BSA (according to Du Bois formula) adjusted for sex, for LV longitudinal strain adjusted for BSA and echocardiography transducer difference, and for EF, LA volume index, LA strain, and MW parameters adjusted for transducer difference.

Re-analyses were done excluding outliers, but this did not significantly affect the results.

To investigate whether the difference in echocardiographic measures between PB/ELBW and controls differed by sex, an interaction term for sex and group affiliation was added.

Comparisons of descriptive variables and outcome data between subgroups of study participants were done by Chi-square test or Fisher`s exact test, Welch´s ANOVA or classic one-way ANOVA (with Games-Howell and Tukey`s test used for post hoc analyses, respectively), or by multiple comparison analyses (with Sidak correction) with the same adjustments as described above, as appropriate.

Pearson correlation coefficient (PCC) was used to explore associations between echocardiographic measured myocardial function and descriptive variables. GA was set to 40 weeks in controls when correlations regarding GA was made.

Inter- and intra-rater reliability were measured by a 2-way mixed-effect model for absolute agreement.

All tests were two-sided, and p < 0.05 was considered statistically significant. All statistical analyses were performed using SPSS version 26.0 (IBM Corp., Armonk, NY, USA).

## Results

The participants consisted of 63 PB/ELBW subjects and 64 age- and sex matches controls. The PB/ELBW group had significantly lower height and weight, with consequently lower BSA, compared to the control group. There was no significant difference for mean age, body mass index, BP, or smoking status between the groups. The age range was 18–37 years in both the PB/ELBW and the control group. BW in the PB/ELBW group ranged from 450 to 1480 g with mean BW 945 (SD 217) grams. GA ranged from 23 to 34 weeks with mean GA 27 (SD 2) weeks (Table 1). None of the study participants had history of cardiovascular disease.

**Table 1:** Characteristics of study participants

	PB/ELBW	Controls	p
Total, n (% males) Cohort 1 (born 1982–1985), n (%) Cohort 2 (born 1991–1992), n (%) Cohort 3 (born 1999–2000), n (%)	63 (43%) 23 (37%) 19 (30%) 21 (33%)	64 (44%) 26 (41%) 18 (28%) 20 (31%)	
GA (weeks), mean (SD)	27 (2)	Term-born	
Birthweight (grams), mean (SD)	945 (217)	3527 (324)	< 0.001^a^
Small for gestational age, n (%)	16 (25%)		
Extremely preterm GA < 28 weeks, n (%)	39 (62%)		
Extremely low birthweight < 1000 g, n (%)	37 (59%)		
Moderate/severe BPD, n (%)	21 (33%)		
Days on ventilator, mean (SD)	8 (9)		
Days on CPAP, mean (SD)	33 (14)		
Days on O_2_-supplement, mean (SD)	52 (35)		
Pre-natal steroid treatment, n (%)	35 (55%)		
Post-natal steroid treatment, n (%)	14 (22%)		
Surfactant, n (%)	28 (44%)		
Age (years), mean (SD)	27 (6)	28 (7)	0.95^b^
Height (cm), mean (95% CI)	168 (167, 170)	174 (172, 175)	< 0.001^c^
Weight (kg), mean (95% CI)	68 (64, 72)	75 (71, 79)	0.01^c^
Smokers, n (%)	4 (6%)	4 (6%)	1.00^b^
BMI (kg/m^2^), mean (SD) BSA (m^2^), mean (95% CI)	24.0 (4.1) 1.8 (1.7, 1.8)	24.9 (6.0) 1.9 (1.8, 1.9)	0.75^b^ < 0.001^c^
Systolic BP (mmHg), mean (SD)	113 (12)	112 (10)	0.87^b^
Diastolic BP (mmHg), mean (SD)	71 (8)	70 (8)	0.98^b^
Heartrate (beats /minute), mean (SD)	69 (14)	65 (11)	0.13^a^

^a = independent samples t test (with equal variance not assumed)^

^b= non−parametric two sample Kolmogorov− Smirnov test^

^c= ANCOVA adjusted for sex^

PB/ELBW: very preterm born/ with extremely low birthweight, SD: standard deviation, GA: gestational age, BPD: bronchopulmonary dysplasia (classified as moderate/severe BPD if need of supplementary oxygen or CPAP at 36 weeks of gestation), CPAP: continuous positive airway pressure; O_2_: oxygen, CI: confidence interval, BMI: body mass index, BSA: body surface area (Du Bois formula), BP: blood pressure, ANCOVA: analysis of covariance

EF, mitral inflow velocities, and annular tissue velocities were measured in most of the participants (Table 2). Estimation of tricuspid valve regurgitation peak gradient was dependent on the presence of a tricuspid valve regurgitation jet and measurable only in 37 PB/ELBW and 41 controls. Measures of LA strain, MW, and LV strain were limited by image quality and compromised speckle-tracking, and lack of adequately measured BP. LV-4 C-LS was assessed in 61 PB/ELBW and 60 controls, LV-GLS in 50 PB/ELBW and 53 controls, MW in 49 PB/ELBW and 51 controls, and LA strain in 55 PB/ELBW and 55 controls.

**Table 2:** Echocardiographic measures of systolic and diastolic myocardial function in PB/ELBW compared to controls

		PB/ELBW		Controls	P
	n		n		
**EF Teicholz** (%), mean (95% CI)	63	61 (59, 62)	64	62 (61, 64)	0.18^a^
**EF Simpson** (%), mean (95% CI)	63	59 (58, 61)	64	61 (59, 62)	0.32^a^
**LV-4 C-LS** (%), mean (95% CI)	61	-18.7 (-19.2, -18.2)	60	-19.9 (-20.5, -19.4)	0.002^a^
**LV-GLS** (%), mean (95% CI)	50	-19.4 (-20.0, -18.9)	53	-20.6 (-21.1, -20.1)	0.003^a^
**GCW** (mmHg%), mean (95% CI)	49	1982 (1901, 2063)	51	2074 (1995, 2153)	0.11^a^
**GWW** (mmHg%), mean (95% CI)	49	100 (78, 121)	51	92 (71, 113)	0.61^a^
**GWI** (mmHg%), mean (95% CI)	49	1749 (1666, 1832)	51	1853 (1772, 1934)	0.08^a^
**GWE** (%), mean (95% CI)	49	95 (93, 96)	51	95 (93, 96)	0.85^a^
**IVSDd** (cm), mean (95% CI)	63	0.9 (0.8, 0.9)	64	0.9 (0.9, 0.9)	0.08^b^
**PWDd** (cm), mean 95% CI)	63	0.8 (0.8, 0.8)	64	0.8 (0.8, 0.9)	1.00^b^
**LVDd** (cm), mean (95% CI)	63	4.6 (4.5, 4.7)	64	4.8 (4.7, 4.9)	0.08^b^
**Indexed LV mass** (g/m^2^), mean (95% CI)	63	69 (65, 73)	64	75 (70, 79)	0.08^c^
**Mitral septal e´**(m/s), mean (95% CI)	61	0.12 (0.11, 0.12)	63	0.12 (0.11, 0.13)	0.99^b^
**Mitral lateral e´** (m/s), mean (95% CI)	62	0.15 (0.14, 0.16)	64	0.16 (0.13, 0.20)	0.96^b^
**Average mitral E/e´ratio**, mean (95% CI)	61	5.5 (5.3, 5.7)	61	5.4 (5.1, 5.8)	0.82^b^
**Mitral E/A-ratio**, mean (95% CI)	63	1.5 (1.4, 1.6)	61	1.5 (1.4, 1.6)	0.83^b^
**TR peak gradient** (mmHg), mean (95% CI)	37	18 (16, 20)	41	16 (15,18)	0.96^b^
**LA contraction strain** (%), mean (95% CI)	55	-10.5 (-11.5, -9.4)	55	-11.5 (-12.5, -10.3)	0.22^a^
**LA conduit strain** (%), mean (95% CI)	55	27.5 (25.5, 29.5)	55	28.6 (26.6, 30.6)	0.43^a^
**LA reservoir strain** (%), mean (95% CI)	55	38.1 (35.7, 40.5)	55	39.7 (37.4, 42.1)	0.34^a^
**LA volume** (ml/m^2^), mean (95% CI)	55	21 (19, 22)	55	21 (19, 22)	0.93^a^

^a = ANCOVA adjusted for BSA and transducer difference as appropriate^

^b= non−parametric two sample Kolmogorov− Smirnov test^

^c = independent samples t test (with equal variance not assumed)^

PB/ELBW: very premature born (< 29 weeks of gestation) or with extremely low birthweight (< 1000 g), EF: ejection fraction, CI: confidence interval, LV: left ventricle, 4 C-LS: 4-chamber longitudinal strain, GLS: global longitudinal strain, GCW: global constructive work, GWW: global wasted work, GWI: global work index, GWE: global work efficiency, IVSDd: intraventricular septum diameter measured in diastole, PWDd: posterior wall diameter measured in diastole, LVDd: left ventricular internal diameter measured in diastole, TR: tricuspid regurgitation, LA: left atrium, ANCOVA: analysis of covariance

### Systolic LV function

**Fig. 4:**
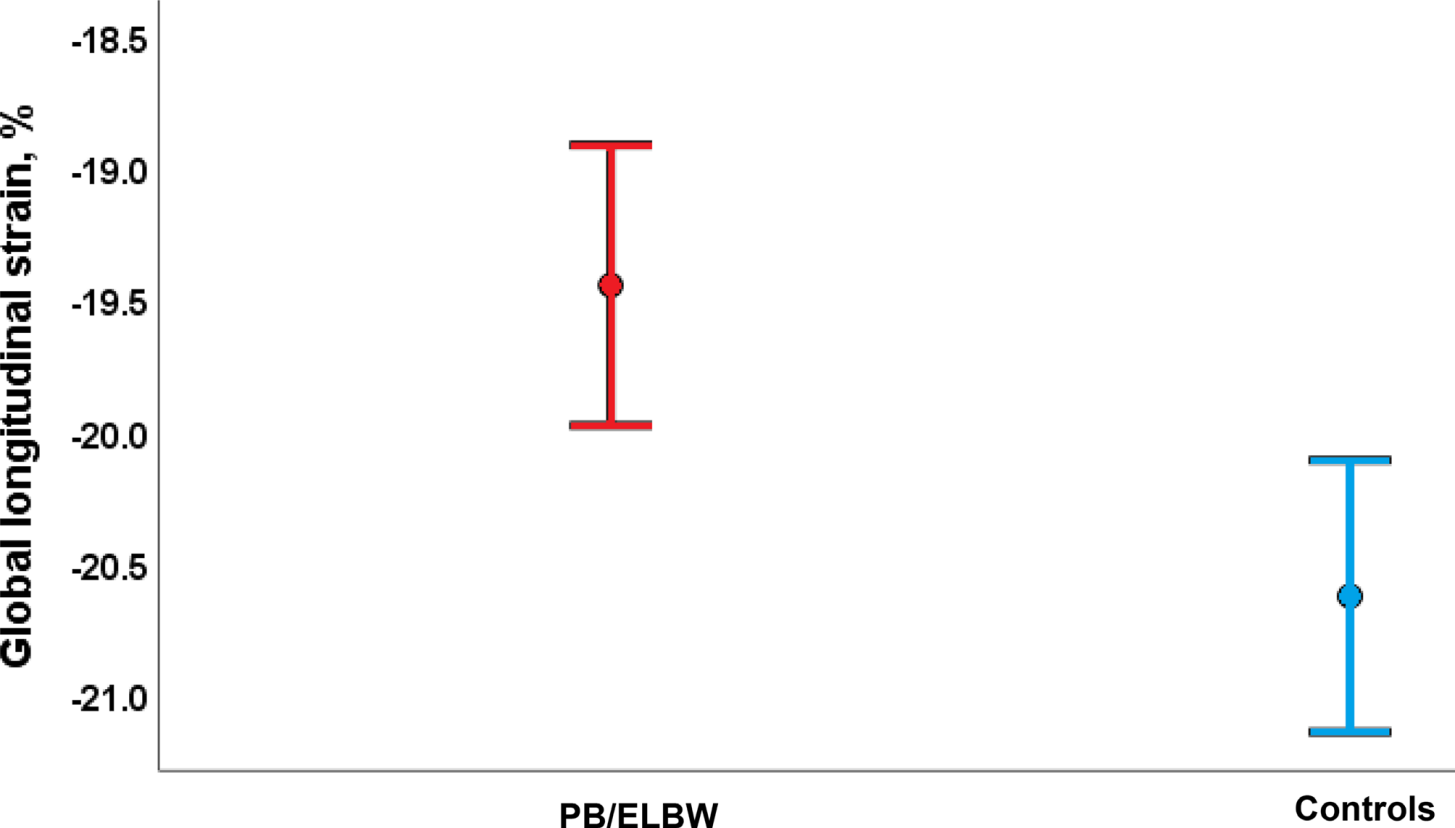
Left ventricular myocardial function measured by peak global longitudinal strain (GLS) was significantly reduced in young adults born very premature or with extremely low birthweight (PB/ELBW) compared to controls. Mean GLS was − 19.4% (95% confidence interval (CI) -20.0, -18.9) in PB/ELBW and − 20.6% (95% CI -21.1, − 20.1) in controls (p = 0.003)

Mean EF Simpson and EF Teicholz were similar in PB/ELBW and controls, but LV-GLS were significantly impaired in PB/ELBW compared to controls − 19.4% (95% CI -20.0, -18.9) versus − 20.6% (95% CI -21.1, -20.1) (p = 0.003) (Table 2; Fig. 4). The difference in LV-GLS between PB/ELBW and controls was not significantly greater in the male participants versus the female participants (1.1% (95% CI -0.3, 2.5) (p = 0.12)).

Lower BW, but not GA was significantly associated with more impaired LV-GLS (PCC − 0.2 with p = 0.02).

Among the PB/ELBW, 4 had reduced EF Simpson, 11 had borderline impaired LV-GLS, and 3 had impaired LV-GLS. Among the controls one had reduced EF Simpson, 6 had borderline impaired LV-GLS, and none had impaired LV-GLS.

### Diastolic LV function

Parameters of LV diastolic function were mainly normal and similar in the PB/ELBW and controls. Of the PB/ELBW, 2 had abnormally low septal e´ velocity, 3 had abnormally low lateral e´ velocity, none had abnormally high average E/e´, 1 had abnormally high tricuspid-valve regurgitation peak gradient, and none had abnormally high LA volume index.

Of the controls, 2 had abnormally low lateral e´ velocity, and 3 had abnormally high LA volume index, otherwise the parameters were normal. None of the participants had more than one abnormal parameter associated with diastolic dysfunction.

**Fig. 5:**
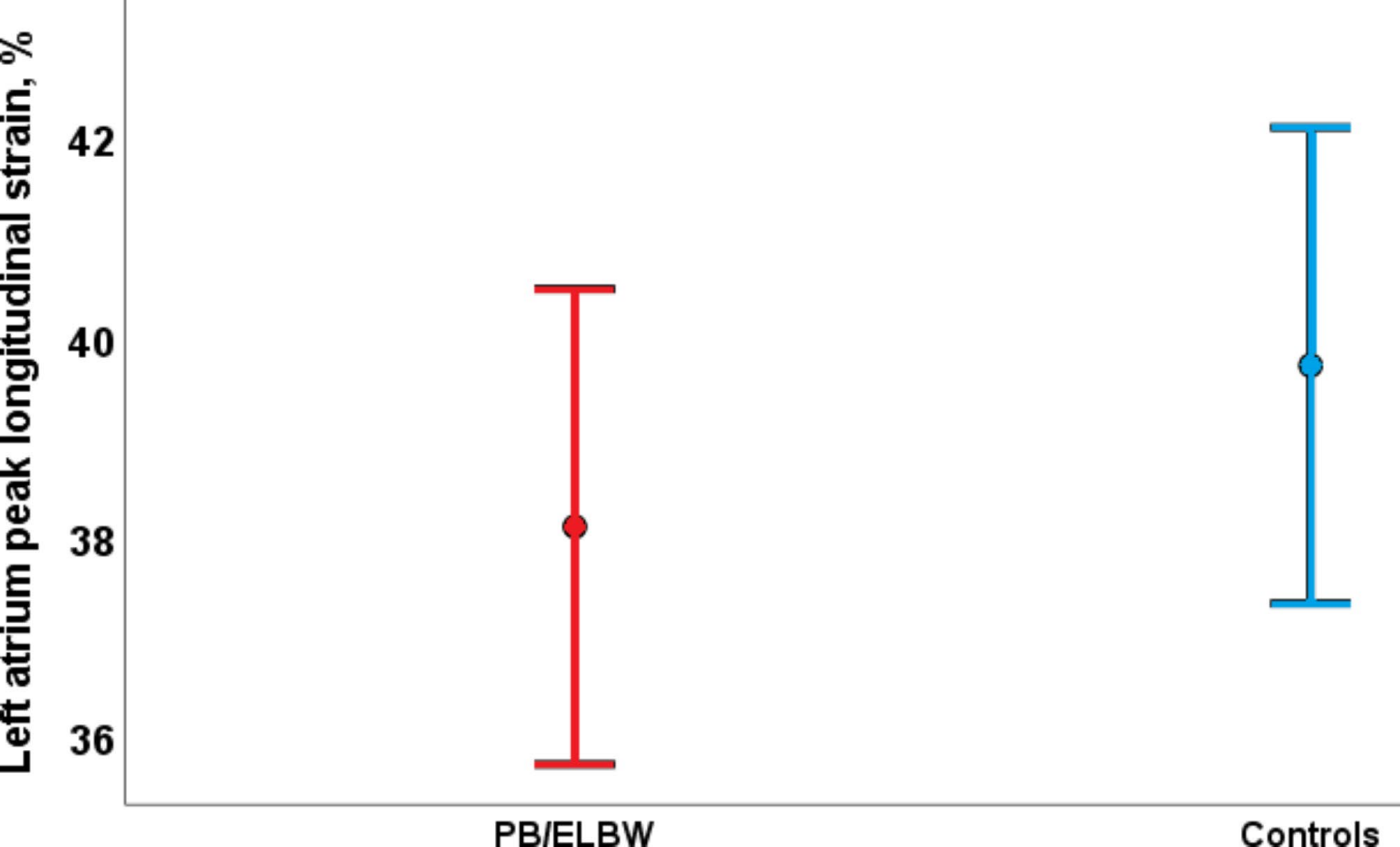
Left atrial reservoir function measured by peak longitudinal strain was similar in young adults born very preterm or with extremely low birthweight (PB/ELBW) and controls. Mean left atrial reservoir strain was 38.1% (95% confidence interval (CI) 35.7, 40.5) in PB/ELBW and 39.7% (95% CI 37.4, 42.1) in controls (p = 0.3)

The mean LA reservoir strain, conduit strain, and contractile strain were similar in PB/ELBW and controls (Table 2; Fig. 5). Lower LA conduit strain, higher average E/e´, lower mitral septal e`, and lower mitral lateral e` were associated to increased chronological age (PCC − 0.2 with p = 0.02, PCC 0.3 with p = 0.001, PCC − 0.5 with p = < 0.01, and PCC − 0.2 with p = 0.01, respectively), but not associated with BW or GA.

LA reservoir strain, conduit strain, and contractile strain were also similar in PB/ELBW and controls when re-analysed while using atrial cycle with zero reference point set at the start of the ECG p-wave.

Myocardial work MW was calculated after determination of LV-GLS and BP in 49 PB/ELBW and 51 controls. Mean LV-GLS was significantly impaired and mean BP was similar in these PB/ELBW compared to these controls. Mean LV-GLS was − 19.4% (95% CI -20.0, -18.9) in PB/ELBW and − 20.6 (95% CI -21.1, -20.1) (p = 0.003) in the controls. Mean BP was 112/69 mmHg (SD 11/7) in PB/ELBW and 111/69 mmHg (SD 11/8) in controls (p = 0.9 and 1.0). One control had systolic BP > 140 mmHg, otherwise BP were below 140/90 mmHg in all participants.

MW is influenced by the contraction power of the myocardial fibres, the LV loading conditions, and the wall stress applied on the LV segments [37]. LV dimension must be taken into consideration since calculation of work would be relatively underestimated in dilated ventricles due to higher wall stress at any given LV pressure than in smaller ventricles [38]. The PB/ELBW and controls had seemingly similar loading conditions and wall stress due to similar means of parameters describing diastolic function and LV filling pressure, BP, heart rate, LV dimension measured by end-diastole internal diameter, and myocardial mass index (Tables 1 and 2).

**Fig. 6:**
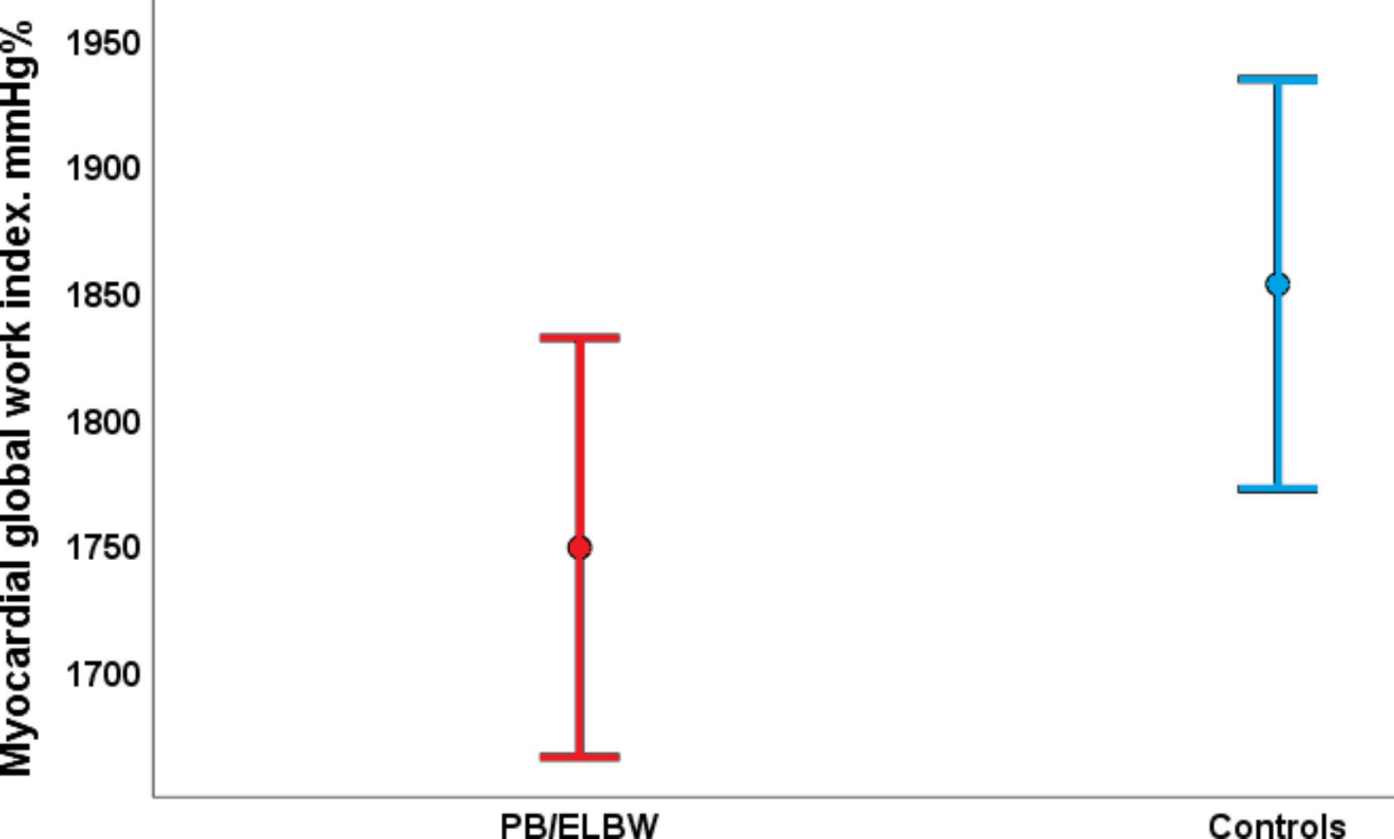
Myocardial performance measured by global work index (GWI) generated from left ventricle pressure-strain loops was similar in young adults born very preterm or with extremely low birthweight (PB/ELBW) and controls. Mean GWI was 1749 mmHg% (95% confidence interval (CI) 1666, 1832) in PB/ELBW and 1853 mmHg% (95% CI 1772, 1934) in controls (p = 0.08)

The MW described as means of GWI, GCW, GWW, and GWE were not significantly different in PB/ELBW and controls, and the measures were close to former described reference values [21]. However, there was a trend towards lower GWI and GCW and higher GWW in PB/ELBW compared to controls (Table 2; Fig. 6).

GWI, GCW, GWW, and GWE were not significantly associated to BW or GA.

Participant characteristics and echocardiographic findings across the three cohorts of PB/ELBW are described in a table in Additional file 2. There were no differences between the cohorts regarding measures of left ventricular systolic and diastolic function, including measures of MW. Additionally, there was no significant associations between treatment in the prenatal and neonatal period (days of oxygen supplement, days of ventilation support, use of surfactant, or steroid treatment) and measures of myocardial function.

## Discussion

In 63 young adults born very preterm or with extremely low BW, we found impaired LV-GLS compared to sex-and age matched controls, but mainly normal systolic function including estimates of MW. Diastolic function including measures of LA longitudinal strain was preserved. Lower BW was associated with more impaired LV-GLS and increasing chronological age was associated with decreasing diastolic function.

Our finding of similar EF in young adults born preterm or with low BW compared to controls, but reduced LV function measured by longitudinal strain, are similar to studies by Huckstep et al. [13] and Lewandowski et al.[14, 15]. However, Goss et al. [18] reported hypercontractile LV function measured by longitudinal strain in 38 young adults at mean age 25 years, born at mean GA 29 weeks. While the studies by Huckstep and Levandowski contained preterm born participants with significantly higher systolic BP and/or higher myocardial mass [13–15], the preterm participants in the study by Goss had lower myocardial mass and similar systolic BP compared to their controls [18]. This may contribute to the difference in result since the study participants otherwise were seemingly similar. Increased LV mass has independently been associated with more impaired LV-GLS in other groups, such as patients with aortic stenosis [39, 40] and athletes [41, 42]. Additionally, strain measurements are known to be afterload dependent with more impaired longitudinal strain with higher BP [32, 38]. Low BW has been classified by the World Health Organization [43] as a risk factor for cardiovascular disease. This corresponds well to our finding of lower BW being associated to more impaired LV-GLS. Although morbidity and all-cause mortality of young adults (15- <50 years) born preterm are reported higher in men compared to women [2], the difference in LV-GLS between PB/ELBW and controls in our study did not differ significantly between sexes.

Similar to our study, Goss et al. [18] reported preserved diastolic function in preterm born young adults. However, several previous studies have found reduced diastolic function in this group. A meta-analysis from Telles et al. [44] and a study by Lewandowski et al.[15] reported an overall lower LV diastolic function in preterm born children and young adulthood as well as indications of increased diffuse myocardial fibrosis. Recently, analysis of LA reservoir strain has been reported to detect increased LV filling pressure and LV diastolic dysfunction at an earlier point than other echocardiographic measures [24, 25]. We found no significant difference between mean LA reservoir strain in PB/ELBW and controls, and a mean LA reservoir strain in PB/ELBW of 38.1% which is close to a formerly described mean reference value (39%) in healthy subjects [36]. These findings support a normal diastolic function in our preterm born young adults. Diverging results between studies may reflect the complex interplay of altered cardiac structure and loading conditions on measures of diastolic function. Aging is associated with reduced LV relaxation, which may lead to diastolic dysfunction [31]. This corresponds well with our finding of a significant association between increased chronological age and lower diastolic function measured by LA conduit strain, E/e´, and mitral septal and lateral e´.

To add further information on myocardial function we estimated MW non-invasively. In subjects with cardiac remodelling and elements of myocardial fibrosis, which has been reported in preterm born young adults [15, 44], we might expect reduced myocardial function with time which can be expressed by decreased GWI and GCW, and increased GWW. Similar to reported findings in subjects with hypertrophic cardiomyopathy [45, 46], we found a trend towards reduced GWI and GCW, and higher GWW, in the PB/ELBW group compared to the controls, although not significantly different.

The additive value of these new parameters describing myocardial function over the traditional ones is still object of study, and further studies are needed to establish clinical utility and prognostic impact. Estimation of MW could add information to distinguish whether reduced contraction patterns are caused by newly emerged hypertension or attenuated contractility, since the increased afterload will reduce EF and LV-GLS, but increase GWI and GCW [38]. This may be especially important in preterm born adults, who have increased prevalence of hypertension [47].

Our study population consisted of three cohorts born in different decades (early 1980s to 2000), in a period with significant changes in the neonatal medical treatment. There was no difference in parameters of left ventricle myocardial function across the cohorts. However, the study samples within the cohorts of the preterm born were too small to reveal minor group differences regarding myocardial function.

There has been an increasing use of prenatal corticosteroids and assisted ventilation during the period, and the use of surfactant was introduced in the early 1990s. Additionally, the techniques have improved, from assisted ventilation mainly by intermittent or continuous positive pressure ventilation via an endotracheal tube in the early period to increasing use of nasal continuous airway pressure and high frequency oscillatory ventilation during the 1990s [48]. The PB/ELBW participants born in 1999–2000 (cohort 3) had significantly lower mean birthweight compared to those born in 1982–1985 (cohort 1) which may indicate increasing survival of the most vulnerable neonates. While the lower mean birthweight in the youngest cohort compared to the oldest cohort (857 g versus 1014 g) could have led to a decreased left ventricular myocardial function in this subgroup, the possible improvement in neonatal intensive care treatment the last decades, and the younger age at the time of assessment, could have the opposite influence on myocardial function in the younger cohorts. To further investigate this, we correlated measures of myocardial function to the treatment in the prenatal and neonatal period (days of oxygen supplement, days of ventilation support, use of surfactant, or steroid treatment), and found no significant associations. Additionally, the measures of myocardial function were similar in the PB/ELBW with moderate to severe bronchopulmonary dysplasia (n = 33) compared to the participants without this diagnosis, and in the PB/ELBW with extremely low BW (n = 37) or extremely preterm birth (n = 39) compared to the remaining PB/ELBW.

## Strengths and limitations

This study is part of a long-term cohort study following individuals born PB/ELBW and individually age and sex matched term-born controls. The study design, and similarity of the PB/ELBW and control group at the time of the assessment reduces the risk of confounding. To diminish procedure related variance the echocardiographic imaging and off-line analyses were performed by one highly trained sonographer using the same ultrasound system and echocardiographic software. The calculated intra- and inter-rater variability were good to excellent [49].

There were limitations to the study. Only 42% of the eligible PB/ELBW subjects were examined. Those who did not participate had similar GA, occurrence of moderate or severe bronchopulmonary dysplasia [50], and days of ventilatory support and oxygen demand, compared to those who did participate (Table 3). The non-participants had a slight and non-significant lower mean BW compared to the participants (896 g versus 948 g, p = 0.1). A possible selection bias could therefore most likely have led to an underestimation of the difference in echocardiographic measures between PB/ELBW and controls since lower BW was associated with more impaired myocardial function.

**Table 3:** Characteristics of participants and non-participants born very preterm or with extremely low birthweight

	Participants (n = 63)	Non-participants (n = 85)	p
Male, n	27 (43%)	47 (55%)	
Cohort 1 born 1982-85, n (%)	23 (37%)	26 (31%)	
Cohort 2 born 1991-92, n (%)	19 (30%)	16 (19%)	
Cohort 3 born 1999–2020, n (%)	21 (33%)	43 (51%)	
BW, grams, mean (SD)	948 (216)	896 (179)	0.1^a^
GA, weeks, mean (SD)	27 (2)	27 (2)	1.0^b^
Days on ventilator, mean (SD)	8 (9)	8 (11)	0.4^b^
Days on CPAP, mean (SD)	33 (14)	29 (20)	0.3^a^
Days on O_2_-supplement, mean (SD)	52 (35)	57 (45)	0.4^b^
Maternal smoking, n (%)	21 (34%) (n = 62)	29 (37%) (n = 77)	1.0^b^
Moderat/severe BPD, n (%)	21 (33%)	37 (44%)	0.8^b^
Pre-natal steroid treatment, n (%)	35 (55%)	48 (56%)	1.0^b^
Post-natal steroid treatment, n (%)	14 (22%)	20 (24%)	1.0^b^
Surfactant, n (%)	28 (44%)	43 (50%)	1.0^b^

^a^= independent sample t test (with variance not assumed)

^b^= non−parametric two sample Kolmogorov− Smirnov test

BW: birthweight; SD: standard deviation; GA: gestational age; CPAP: continuous positive airway pressure; O_2_: oxygen; BPD: bronchopulmonary dysplasia

Only four chamber view images were used for analyses of LA strain while reference values and guidelines are based on measures of two- and four chamber view images. The mean values for LA strain might therefore deviate from reference values, but would not affect the analysis of group difference between PB/ELBW and controls. However, the mean LA peak longitudinal strain in our control group (39.7%) was similar to former reported reference values in healthy subjects (39%). Although R-R gated LA strain analysis is recommended in the guidelines and more frequently used, P-P gating has been found to correlate better to new-onset heart failure and to 3-dimentional echocardiographic-derived measures [51, 52]. LA strain was re-analysed using P-P gating with zero reference point set at the start of the ECG P-wave. As expected, this generated lower LA peak longitudinal strain values (reservoir strain) [36], but did not change the main result when mean LA strain values in PB/ELBW and control were compared.

LV-GLS, but not estimates of MW, were found impaired in PB/ELBW compared to controls. The study was not designed to prove the superiority of MW to LV-GLS, and although analyses of MW have been considered an advancement of LV strain analyses due to reduction of the strain analysis` load dependent limitations [20, 21], the method cannot be considered load-independent, as it is derived from strain measurements. Larger scale studies are needed to establish the clinical utility and prognostic impact of MW on cardiovascular outcome in preterm born young adults.

## Conclusion

Young adults born very preterm (< 29 weeks of gestation) or with extremely low BW (< 1000 g) had impaired LV-GLS compared to term-born controls, although mainly normal systolic function. Myocardial function measured by MW and diastolic function were preserved. Lower BW was associated with more impaired LV-GLS and increased chronological age was associated with decreasing diastolic function. These findings could indicate an elevated risk in preterm born young adults of developing heart failure. Given the increasing survival rate of preterm born neonates it is of public health interest to generate prevention strategies to reduce modifiable risk factors and to implement long-term cardiovascular follow-up in this group. Further studies are needed to investigate the impact of preterm birth and low BW on myocardial function in young adulthood to optimize risk stratification.

## Electronic supplementary material

Below is the link to the electronic supplementary material.

Additional File 1: Flow chart of the study population

Additional File 2: Characteristics and echocardiographic measures of participants born very preterm/with extremely low birthweight by cohort

## Data Availability

In accordance with the approvals granted for this study by The Regional Committee on Medical Research Ethics and The Norwegian Data Protection Authority, the data files are stored securely and in accordance with the Norwegian Law of Privacy Protection. The data file cannot be made publicly available as this might compromise the respondents’ privacy. Some of the participating centres are small and the number of extremely preterm births limited with a risk of identifying anonymous participants. To prepare future research papers other researchers in our group currently use the data file. A subset of the data file with anonymized data can be made available to interested researchers upon reasonable request to Maria Vollsæter (maria.vollseter@helse-bergen.no), providing Norwegian privacy legislation and GDPR are respected, and that permission is granted from The Norwegian Data Protection Authority and the data protection officer at Haukeland University Hospital.

## Abbreviations

AFI: automated function imaging
ANCOVA: analyses of covariance
BP: blood pressure
BPD: bronchopulmonary dysplasia
BSA: body surface area
BW: birthweight
CL: confidence interval
CPAP: continuous positive airway pressure
EF: ejection fraction
GA: gestational age
GCW: global constructive work
GLS: global longitudinal strain
GWE: global work efficiency
GWI: global work index
GWW: global wasted work
LA: left atrial
LV: left ventricle
LV-4C-LS: left ventricle longitudinal strain from the apical four chamber view
MW: myocardial work
O_2_: oxygen
PCC: Pearson correlation coefficient
PB/ELBW: very preterm born or with extremely low birthweight
ROI: region of interest
SD: standard deviation
