# Supplementary material for: Myocardial function including estimates of myocardial work in young adults born very preterm or with extremely low birthweight - a cohort study
Source: BMC Cardiovasc Disord. 2023 Apr 29;23:222. doi: 10.1186/s12872-023-03253-4 (PMC10149027; doi:10.1186/s12872-023-03253-4)
Supplement: Supplementary file 1 — Additional File 1: Flow chart of the study population [file 12872_2023_3253_MOESM1_ESM.pdf]

Additional file 1

Flow chart of the study population

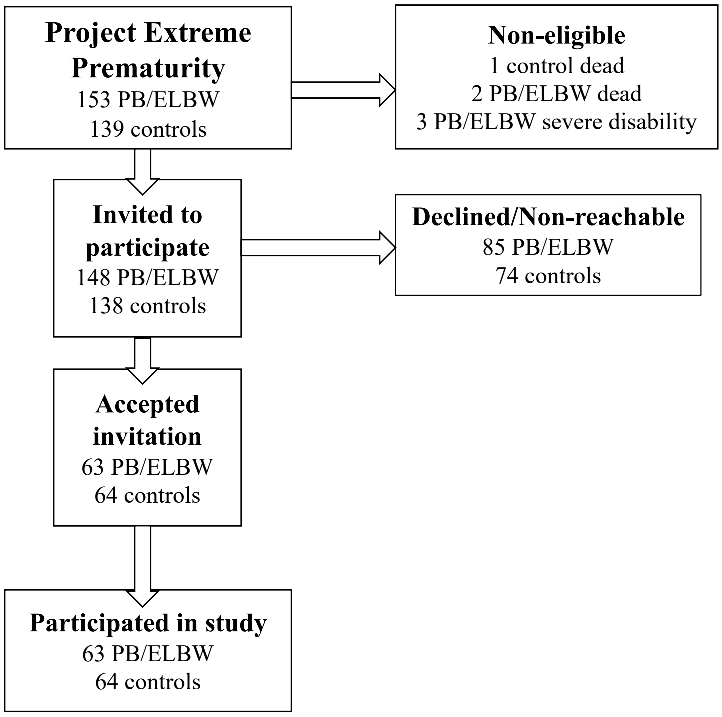

Flow chart of the study population in the study of left ventricular myocardial function in young adults born very preterm or with extremely low birthweight (PB/ELBW) and term-born controls, part of the Project Extreme Prematurity in Norway.
