# Supplementary material for: Myocardial function including estimates of myocardial work in young adults born very preterm or with extremely low birthweight - a cohort study
Source: BMC Cardiovasc Disord. 2023 Apr 29;23:222. doi: 10.1186/s12872-023-03253-4 (PMC10149027; doi:10.1186/s12872-023-03253-4)
Supplement: Supplementary file 2 — Additional File 2: Characteristics and echocardiographic measures of participants born very preterm/with extremely low birthweight by cohort [file 12872_2023_3253_MOESM2_ESM.pdf]

Additional file 2

Characteristics and echocardiographic measures of participants born very preterm/with extremely low birthweight by cohort

	Cohort I born 1982-85 n=23	Cohort II born 1991-92 n=19	Cohort III born 1999-2000 n=21	p
**Characteristics**				
Age (years), mean (SD)	34 (1.5)	27 (0.6)	19 (0.8)	<0.001[a]
Males, n (%)	11 (48%)	7 (37%)	9 (43%)	0.74[b]
BW (grams), mean (SD)	1014 (210)	959 (225)	857 (194)	0.05[c]
GA (weeks), mean (SD)	28 (2)	27 (2)	27 (1)	0.13[c]
Extremely preterm GA <28 weeks, n (%)	11 (48%)	11 (58%)	17 (81%)	0.07[b]
Small for gestational age, n (%)	4 (17%)	4 (21%)	8 (38%)	0.29[b]
Extremely BW <1000 g, n (%)	9 (39%)	11 (58%)	17 (81%)	0.02[b]
Moderate/severe BPD, n (%)	6 (26%)	8 (42%)	7 (33%)	0.55[b]
Days O_2-supplement, mean (SD)	44 (21)	61 (51)	53 (25)	0.25[a]
Days on ventilator, mean (SD)	10 (10)	8 (10)	6 (5)	0.30[b]
Days on CPAP, mean (SD)	0	0	33 (14)	
Surfactant, n (%)	0	9 (47%)	19 (90%)	<0.001[b]
Pre-natal steroid treatment, n (%)	8 (35%)	7 (37%)	20 (95%)	<0.001[b]
Post-natal steroid treatment, n (%)	1 (4%)	6 (3%)	7 (33%)	0.03[b]
BMI (kg/m2), mean (SD)	24.7 (4.0)	24.7 (4.2)	22.6 (4.0)	0.12[c]
BSA (m2), mean (95% CI)	1.8 (1.7, 1.8)	1.8 (1.7, 1.9)	1.7 (1.7, 1.8)	0.16[d]
Height (cm), mean (95% CI)	168 (165, 170)	169 (167, 171)	168 (166, 170)	0.74[d]
Weight (kg), mean (95% CI)	69 (64, 74)	71 (66, 77)	64 (59, 69)	0.13[d]
Smokers, n	2	1	0	
Systolic BP (mmHg), mean (SD)	112 (13)	114 (13)	112 (10)	0.72[c]
Diastolic BP (mmHg), mean (SD)	73 (10)	71 (6)	68 (8)	0.15[c]
**Echocardiographic measurements**				
Indexed LV mass (g/m2), mean (95% CI)	70 (64, 77)	70 (62, 78)	66 (58, 75)	0.65[c]
EF Simpson (%), mean (95% CI)	60 (57, 61)	60 (57,63)	59 (56, 62)	0.61[d]
LV-GLS (%), mean (95% CI)	-20.2 (-21.0, -19.5)	-20.0 (-20.7, -19.3)	-19.9 (-20.8, -19.1)	0.86[d]
GCW (mmHg%), mean (95% CI)	1885 (1719, 2051)	2101 (1943, 2259)	1934 (1748, 2120)	0.13[d]
GWW (mmHg%), mean (95% CI)	85 (30, 141)	124 (73, 177)	88 (26, 150)	0.50[d]
GWI (mmHg%), mean (95% CI)	1667 (1484, 1849)	1847 (1673, 2020)	1709 (1504, 1913)	0.30[d]

GWE (%), mean (95% CI)	95 (93, 97)	94 (92, 96)	95 (92, 98)	0.82[d]
Mitral septal e´(m/s), mean (95% CI)	0.10 (0.09, 0.11)	0.11 (0.10, 0.13)	0.13 (0.11, 0.12)	0.001[b]
Mitral lateral e´ (m/s), mean (95% CI)	0.14 (0.12, 0.15)	0.15 (0.13, 0.16)	0.17 (0.15, 0.18)	0.012[b]
Average mitral E/e´ratio, mean (95% CI)	5.6 (5.2, 5.9)	5.7 (5.2, 6.2)	5.2 (4.9, 5.7)	0.35[b]
Mitral E/A-ratio, mean (95% CI)	1.3 (1.1, 1.4)	1.5 (1.3, 1.7)	1.7 (1.4, 1.9)	0.02[b]
TR peak gradient (mmHg), mean (95% CI)	19 (13, 24)	16 (13, 18)	19 (16, 20)	0.17[a]
LA contraction strain (%), mean (95% CI)	-12 (-14, -10)	-11 (-13, -9)	-9 (-11, -7)	0.34[d]
LA conduit strain (%), mean (95% CI)	26 (22, 30)	27 (22, 31)	30 (25, 34)	0.55[d]
LA reservoir strain (%), mean (95% CI)	38 (33, 43)	38 (33, 43)	38 (33, 44)	0.98[d]
LA volume (ml/m2), mean (95% CI)	22 (19, 25)	21 (18, 24)	20 (17, 23)	0.58[d]

a= Welch´s ANOVA, post hoc Games-Howell

b= Chi-square test or Fisher`s exact test

c= classic ANOVA, post hoc Tukey

d= ANCOVA (with Sidak correction) adjusted for sex, BSA, and echocardiographic transducer difference as appropriate.

SD: standard deviation, BW: birthweight, GA: gestational age, BPD: bronchopulmonary dysplasia (classified as moderate/severe BPD if need of supplementary oxygen or CPAP at 36 weeks of gestation), O_2: oxygen, CPAP: continuous positive airway pressure, BMI: body mass index, BSA: body surface area (Du Bois formula), CI: confidence interval, BP: blood pressure, EF: ejection fraction, LV-GLS: left ventricle global longitudinal strain, GCW: global constructive work, GWW: global wasted work, GWI: global work index, GWE: global work efficiency, TR: tricuspid regurgitation, LA: left atrium, ANOVA: analysis of variance
